# Supplementary material for: Chromothripsis during telomere crisis is independent of NHEJ, and consistent with a replicative origin
Source: Genome Res. 2019 May;29(5):737–49. doi: 10.1101/gr.240705.118 (PMC6499312; doi:10.1101/gr.240705.118)
Supplement: Supplemental Material [file supp_gr.240705.118_Supplemental_file_1.zip › contigs/annotated_contigs/DB51/contig.2.DB51_length_706_mean_cov_9.13597733711.docx]

**DB51_length_706_mean_cov_9.13597733711**

```
AAATTATGACTTAATCTTGAATATACTTCTCGTAAAT|ATCATATATAGAAATGGGATATGAATTAACAGAAAATTATTTTCAATATTC
                                      >chr14:29061637-29061934 - E=7e-155
AATGTTATAGAAATGTCTATGCTTATTGACATTTTTGGTAAGAATTTTTGTTAATAAAATGCATTTGGGTTGCTACTTAACTGATTGTG

AATTAACATTATTTGTGTTATAGAATGAATTGGAAAAGCAAATACTTCTGTGTTCTACTGTCAAATGTGATGCATTCAAATAAGGGAAG

TTATGACAGAAAAAACAAATCTTCCTCAACTTGTCCACATCAGTTCCATTCCCTCATTTATAGGGA|AG|TTTGTTCAAATAATTTAGT
                                                                   >chr14:29060683-2906
CAGGATTTGCATACTTAGCATGATTGACTCTGAAGTGAGTTATTGGGCAGTGCCTGGCATTCAATCAGTAACACCTGAGGAGAACATTG
1057 - E=3e-213
CCTTTGTGTTGCAACCAAGAAGAAGTTAAAAAAGGCACAGAGATGAGGGATCTTTTTTTTATAAGGGGAATTCTGATCATTCTTCTCTG

ATCTTGTTTTCAACTTGTGTCTTAATTTCTTCATATTTGATGCCTCAAGTTAATAGGATTTGTATTGCCATAGGCACTATTGCCATTTC

ATATGCTGTATTAGGGAAGCGGAGTTGCTGATTAGAAAAGTGATAATGCAACTTCCTCTTCTTTCTAAATTAACCTTGGAGTGGAA
```
